# Association of Heavy Rainfall on Genotypic Diversity in *V. cholerae* Isolates from an Outbreak in India

**DOI:** 10.1155/2011/230597

**Published:** 2011-12-12

**Authors:** A. K. Goel, S. C. Jiang

**Affiliations:** ^1^Biotechnology Division, Defense Research & Development Establishment, Gwalior 474 002, India; ^2^Department of Civil & Environmental Engineering, University of California, Irvine, CA 92697, USA

## Abstract

The outbreak of waterborne disease cholera has been associated with rainfall and flooding events by contamination of potable water with environmental *Vibrio cholerae*. The continuation of the epidemic in a region, however, is often due to secondary transmission of the initial outbreak strain through human waste. This paper reports, on the contrary, a rapid shift of genotype from one *V. cholerae* strain to another one in an epidemic region. *V. cholerae* isolated from patients during 2005 cholera epidemic in Chennai, India were characterized using PCR identification of toxin genes, antibiogram, and genomic fingerprinting analysis. The results showed that in spite of the similarity of toxin genes and antibiogram, the *Vibrio* isolates grouped into two different clusters based on the ERIC-PCR fingerprinting. Each cluster corresponded to a distinct peak of cholera outbreak, which occurred after separate heavy rainfall. The results suggest that the rainfall event can bring various genotypes of *V. cholerae* strains causing multiple outbreaks.

## 1. Introduction

Cholera is a well-known waterborne disease for its epidemic and pandemic potentials. Several epidemics and sporadic cases of cholera are reported from many regions of the world every year [1, 2]. During an outbreak, cholera spreads by faecal contaminated water and is influenced by other socio-economical factors such as lack of proper sanitary systems and poor health care [3].

Cholera epidemics are cyclical on the Indian subcontinent [4]. The seasonal recurrence of cholera has been observed since the beginning of the 20th century in Chennai (previously known as Madras Presidency), India [5]. More recently, an increasing number of papers reported the climatic influence on cholera epidemic patterns on the Indian subcontinent. More specifically, cholera epidemics are correlated with seasonal precipitation, wet to dry period, sea surface temperature, and sea level height [4, 6–8]. The link between local climate and cholera epidemic is also supported by the observations that epidemic cycles are different in separate geographical regions. Two peaks of cholera are observed annually in Bangladesh, whereas in south India only one peak is observed and is associated with the rain [4, 9, 10].

Despite the clear association between the seasonal rainfall and cholera, the source of the disease agents that initiates an outbreak is less obvious. It is known that *V. cholerae* are inhabitants of coastal environments [7]. Two routes have been suggested for dissemination of cholera among humans [11]. The first route, the route of primary transmission, links the environment with drinking water and food, while the second route, the route of secondary transmission, is through human faecal contamination of water and food. However, it is unclear whether the seasonal epidemic arises from a single clonal strain through secondary transmission or reflects superimposition of multiple small outbreaks. Stine et al. [12] concluded that seasonal cholera epidemics do not arise from a single clonal strain in Bangladesh; yet, the strain was associated with the individual geographical sampling location. Recent molecular epidemiological investigations have shown genotypic variability among *Vibrio cholerae* O1 or O139 strains in Asia and Africa on temporal and geographical scales [13, 14]. Here, based on molecular epidemiological results, we report a rapid shift of genotype among *V. cholerae* outbreak strains isolated from south India in November 2005. This genotype shift is associated with the excessive heavy rainfall, suggesting that *V. cholerae* transmission from environmental sources is responsible for two separate cholera peaks during the same cholera season at the same location.

**Description of Study Area**

**Figure 1:**
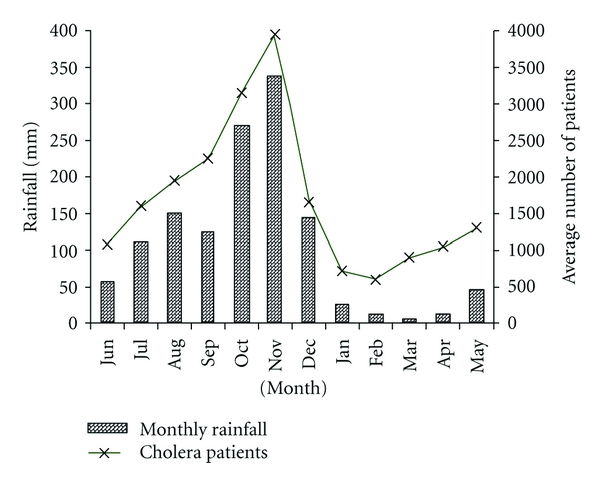
Historical records of seasonal rainfall and the monthly cholera diarrheal cases in Tamil Nadu, India. Bars represent rainfall and line represents monthly average of cholera patients.

Chennai, the location where we focused this study, is a major metropolitan city adjacent to the Bay of Bengal and is located in Tamil Nadu, a major state in south India. The climate in the region has four seasons. There are two rainy seasons each year; one due to North East Monsoon (NEM) occurs between October and December and the other due to South West Monsoon (SWM) during June and September. NEM season marks the highest monthly precipitation of the year, average ~400 mm rainfall.  Figure 1 shows the seasonal rainfall data retrieved from Meteorological Department, Chennai, India (http://www.tn.gov.in/crop/rainfall.htm).

NEM in Tamil Nadu is also characterized with the onset of cholera season each year. Based on data provided by the Communicable Disease Hospital, Chennai, cholera cases in the region start to climb at the end of September and peak in November followed by a decline in December. A 15-year monthly average of cholera cases between 1982 and 1996 indicated that monthly cholera cases reached 4000 cases in Tamil Nadu within the month of November (Figure 1).

**Figure 2:**
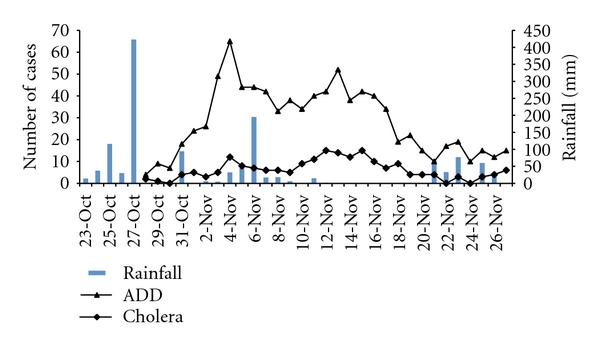
Daily number of patients with acute diarrheal disease (ADD) including cholera and confirmed daily number of cholera cases admitted to Communicable Disease Hospital, Chennai, India, between Oct 27 and Nov 26, 2005. The daily rainfall is overlaid with the illness cases as bar graph. The first peak of illness was observed eight days after the first heavy rain storm on Oct. 27th. The second illness peak was found seven days after the second major rain storm on Nov. 6th.

In year 2005, the record-high level of rain was observed during NEM in Chennai. The monthly rainfall was ~300% above the average in the region during the month of October. The first heavy precipitation of the season was observed on 27th of October 2005 (Figure 2), recording 423 mm in a day (http://www.kea.metsite.com/Monthly_Summaries.htm), the second highest daily rainfall record in history (http://www.kea.metsite.com/rainfall.htm). The first confirmed case of cholera was recorded 3 days after the rain at Communicable Disease Hospital, Chennai, the largest governmental funded hospital in the city (Figure 2). In the following week, acute diarrheal disease (ADD) cases climbed rapidly to 70 cases per day on the 3rd of November before starting a gradual decline (Figure 2). However, the trend of decline was reversed following the second rainstorm on the 6th of November. The second peak of diarrhoea was observed 7 days after the rain (Figure 2) in the hospital. The daily number of ADD reached 55 per day on the 12th of November 2005. Only a subset of diarrheal patients were tested for cholera in the hospital, and all tests were confirmed to be positive for *V. cholerae*, suggesting that a large portion of diarrheal cases were due to cholera during the period of outbreak.

The onset of cholera outbreak in late October 2005 followed the regular epidemic cycle in the region. It was likely due to the contamination of drinking water with environmental *V. cholerae* strains when river and coastal area became flooded due to the heavy rainfall [7]. However, the distinct peaks of cholera were not common in the previous years. To investigate the two peaks of cholera in the month of November and the possible source of the cholera bacteria, we molecularly characterized the *V. cholerae* strains isolated from the two outbreaks. We hypothesized that the *Vibrio* strains that caused the secondary outbreak is a clone of the strain that caused the initial outbreak.

## 2. Materials and Methods

### 2.1. Bacterial Cultures

Stool samples were collected using sterile rectal swabs from symptomatically selected patients that admitted to the Communicable Disease Hospital, Chennai, India in late October and November 2005 [15]. Bacteria were isolated on TCBS agar (Difco, USA). The first confirmed *V. cholerae* isolate was obtained from the sample collected on November 1st, 2005. One to three fresh stool samples were screened each day, and a total of 22 isolates were collected between November 1st and November 18th. Reference strains of *Vibrio cholerae* O1 and O139 were obtained from the American Type Culture Collection (ATCC) and the Indian National Institute of Cholera and Enteric Diseases (NICED), respectively.

### 2.2. Biochemical and Serological Characterization

All bacterial isolates were screened for oxidase reaction test followed by presumptive identification of *V. cholerae *as previously described [15]. Serological identification of the isolates was done by slide agglutination using commercially available polyvalent antiserum against *Vibrio cholerae *O1 and O139 (Difco, USA).

### 2.3. Antibiotic Susceptibility

Antibiotic susceptibility of the *V. cholerae *isolates was determined by the disk diffusion method on Mueller Hinton agar as described earlier [16, 17]. The following antibiotic disks, procured from HiMedia, India were used in the study: ampicillin (10 *μ*g), chloramphenicol (30 *μ*g), ceftazidime (10 *μ*g), ciprofloxacin (5 *μ*g), co-trimoxazole (25 *μ*g), gentamicin (10 *μ*g), kanamycin (30 *μ*g), nalidixic acid (30 *μ*g), nitrofurantoin (300 *μ*g), norfloxacin (10 *μ*g), ofloxacin (5 *μ*g), polymyxin-B (300 *μ*g), rifampicin (5 *μ*g), streptomycin (10 *μ*g), tetracycline (30 *μ*g), and trimethoprim (5 *μ*g). The isolates were scored as resistant or sensitive according to the manufacturer's instructions.

### 2.4. PCR Detection of Gene Traits

Genomic DNA was extracted from each of the isolates using genomic DNA purification kit (Fermentas, Vilnius, Lithuania). The purity of DNA was assessed spectrophotometrically (NanoDrop, USA). All isolates were screened for the presence of genetic traits of *V. cholerae, *including *ompW*, *rfbO1. ctxB*, *zot*, *ace*, *tcp*, *hlyA*, and *toxR*, by PCR using gene-specific primers [18]. Oligonucleotide sequences and PCR conditions are identical as described earlier [18].

### 2.5. Sequencing of Cholera Toxin (ctxB) Gene

Cholera toxin B (*ctxB*) gene was amplified from the isolates using the *ctxF* and *ctxR* primers as previously described [19]. The PCR product was purified and submitted to MWG-Biotech Pvt. Ltd., India for sequencing using ABI PRISM automatic sequencer (Model 3730, USA). The nucleotide sequences obtained were aligned with the *ctxB *gene sequences of classical and El Tor strains (GenBank). The nucleotide sequence obtained for the *ctxB* gene of strains VCM5, VCM7, VCM9, VCM10, VCM11, VCM16, VCM21, VCM24, VCM29, VCM30, VCM32, and VCM35 has been deposited in GenBank under accession numbers *EU496260*, *EU496261*, *EU496262*, *EU496263*, *EU496264*, *EU496265*, *EU496266*, *EU496267*, *EU496268*, *EU496269*, *EU496270*, and *EU496271*, respectively.

### 2.6. Genomic Fingerprinting by ERIC-PCR

Enterobacterial repetitive intergenic consensus (ERIC) sequence PCR was performed by using two oligonucleotides ERIC1R (5′-ATGTAAGCTCCTGGGGATTCAC-3′) and ERIC2 (5′-AAGTAAGTGACTGGGTGAGCG-3′) as described earlier [18]. Digitized fingerprints were analyzed using GelCompar II (Applied Maths, Sint-Martens-Latem, Belgium) software, following the manufacturer's instructions. The clustering method of Ward, which uses an analysis of variance approach to evaluate the distances between clusters and is generally applied to gel pattern analysis, was used to create the dendrogram.

## 3. Results and Discussion

The biochemical and serological tests showed that all isolates collected from diarrheal patients (VCM1–VCM38) in Communicable Disease Hospital, Chennai were *V. cholerae *O1 El Tor Ogawa. All the isolates exhibited resistance towards cotrimoxazole, nalidixic acid, nitrofurantoin, streptomycin, and polymyxin B but were susceptible towards the other antibiotics used in study. There was no variation in the antibiogram profile among the isolates from the first and the second illness peak. PCR analysis further confirmed that all strains were positive for genes specific to *V. cholerae* (*ompW), *somatic antigen depicting serotype O1 *(rfbO1)*, and other toxin and regulatory genes (*ctxB*, *zot*, *ace*, *tcp*, *hlyA*, and *toxR) *regardless of the date of isolation. Nucleotide sequences of *ctxB *gene from these El Tor strains revealed the presence of *ctxB *of classical biotype. This result confirmed several earlier reports indicating that El Tor strains with classical *ctxB* gene have replaced the original El Tor strain in many regions of the world [18, 20–23].

**Figure 3:**
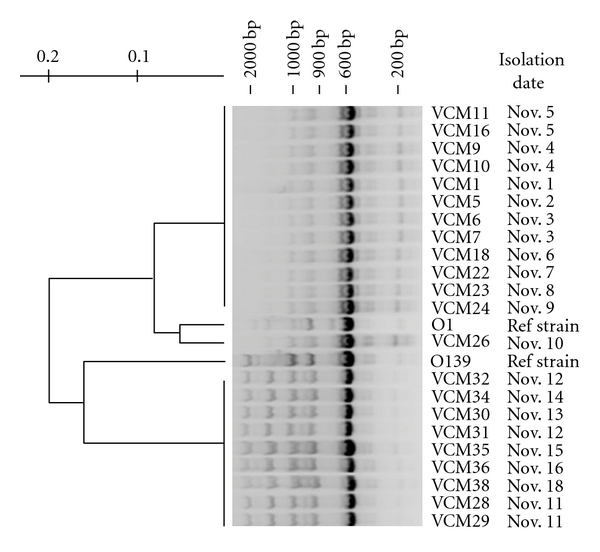
Cluster analysis of ERIC-PCR fingerprints of *V. cholerae *isolates in November 2005. Isolates were grouped into two clusters, Genotype1: VCM1–VCM24; and Genotype2 VCM28–VCM38, that were separated by the date of isolation. *V. cholerae *O1 (ATCC 14033) and O139 (NICED) were used as references strains.

Genomic fingerprinting analysis by ERIC-PCR showed amplification of multiple fragments of DNA ranging between 0.15 and 1.8 kb in size (Figure 3). Isolates obtained from patients admitted to hospital between November 1st to November 9th (VCM1–VCM24), corresponding to the first cholera peak, had identical fingerprinting pattern (Genogroup 1), while isolates obtained between November 11th and November 18th (VCM28–VCM38), corresponding to the second cholera peak, were also nearly identical in ERIC-PCR pattern (Genogroup 2). Cluster analysis, thus, grouped the isolates into two groups according to the date of isolation (Figure 3). The first group (Genogroup 1) is >90% similar to O1 type strain while the second group (Genogroup 2) is more similar to O139 type strain and is >20% different from the first group. Although the variability in fingerprinting pattern among the clonal strains is known [24], the >20% difference between the two groups suggests that *Vibrio* isolates that caused the second cholera peak are not the same strain as the one responsible for the first cholera peak. We are further convinced that the strains isolated from the patients admitted to hospital after November 11 are not the same clonal strain that caused the first cholera peak based on the fact that Genogroup 1 is more similar to reference O1 type strain than to the later isolates. It is also interesting to observe that one of the isolate, VCM26, obtained from a patient admitted on Nov. 10 is different from either group. Thus, there may be additional groups or transitional groups of *V. cholera* that have not been captured by this study. Furthermore, both genotypes were different from isolates collected from the same region a year early (2004). However, the Genotype 1 was more similar to the 2004 isolates presented in an earlier report [25].

Fingerprinting analysis has been widely applied in recent years for *V. cholerae* research to understand the molecular epidemiology of cholera disease [26]. This technique could resolve genotypic variability among cholera strains from different regions [27, 28] and seasonal genotype successions among environmental *V. cholerae* in the aquatic environment [29]. Although only 22 clinical isolates were genotyped in this study, these isolates were selected from random patients admitted to the hospital. The identical fingerprinting pattern observed from the isolates, thus, suggests that the sample size is sufficient to capture the genotypic variability among patients. This study, therefore, showed a short-term shift in *V. cholerae* genotype within two weeks among disease population and that the second illness peak was not a simple continuation of the first epidemic. The transmission of *V. cholerae* from the environment to human was the likely route that resulted in the onset of the second outbreak. Although the high diversity of the environmental *V. cholerae* challenges the matching of environmental strains with clinical isolates, it is plausible that a new environmental strain was introduced into the drinking water system following the second heavy rainfall. The rainstorm and flooding were responsible for the mixing of river and coastal water with drinking water. In most likelihood, two clones of *V. cholerae* were present in the city during the two-week period. One clone became dominant after the first heavy rainfall, whereas the second one was introduced after the second rain event.

The outcome of this study is different from another study conducted in the same region in 2004 [25]. In the early study, it has been shown that all *V. cholerae* isolates collected over a seven-month period had identical fingerprinting profile, suggesting that all isolates were derived from the same endemic strain. Comparing the 2004 with the 2005 study, the most significant difference was the level of rainfall and number of cholera cases. NEM in 2004 had lower than average rainfall in November and December, and cholera epidemic in the community persisted at constant but low level, while the 2005 monsoon was marked by excessive heavy precipitation record and two major pulses of cholera in the community. The rapid change in *V. cholerae* genome type among cholera patients suggests the significant influence of heavy rainfall on genotypic diversity among outbreak strains and different source of disease agents. In summary, this study reports that separate heavy rainfall events could introduce different genotypes *V. cholerae* in the affected area, and this scenario should be taken into consideration as another variable in the management of cholera outbreak.
